# Understanding the metabolic perturbations in *Carica papaya* Linn. due to different ripening practices/agents using gas chromatography‐mass spectrometry based metabolomics

**DOI:** 10.1002/ansa.202000026

**Published:** 2020-07-16

**Authors:** Sireesha Ganneru, Hussain Shaik, Kiranmayi Peddi, Mohana Krishna Reddy Mudiam

**Affiliations:** ^1^ Department of Biochemistry Acharya Nagarjuna University Nagarjuna Nagar India; ^2^ Analytical and Structural Chemistry Department CSIR‐Indian Institute of Chemical Technology Hyderabad India; ^3^ Academy of Scientific & Innovative Research (AcSIR) CSIR‐IICT Campus Hyderabad India

**Keywords:** calcium carbide, ethylene, metabolomics, Papaya (*Carica papaya* Linn.), ripening agents

## Abstract

The study of fruit‐ripening mechanism is vital as it plays a key role in the maintenance of fruit quality. Use of various xenobiotics for quick ripening has been shown to impact the quality of fruit, which in turn affect human health. In the present study, we made an attempt to understand the metabolic perturbations in *Carica papaya* Linn. (papaya), which has been ripened either by the ripening practice (room temperature process as control) and/or ripening agents (calcium carbide and ethylene) using gas chromatography‐mass spectrometry (GC‐MS) based metabolomics. The partial least squares‐discriminant analysis has revealed significant alternations in 13 metabolites mainly sugars, amino acids, fatty acids, and organic acids as well as disturbances in five metabolic pathways due to different ripening practice/agents. The individual comparison of calcium carbide with control and ethylene with control has found 13 and 11 metabolites, respectively, which are common to the PLS‐DA of three ripening groups. The GC‐MS–based metabolomics has been able to predict the metabolic perturbations in papaya resulting from the ripening practice/agents. The findings from the present analysis has a wide application in food quality and will help to address safety concerns.

**Abbreviations**
AUROC: area under receiver operating characteristics
MetPA: metabolic pathway analysis
PCA: principal component analysis
PLS‐DA: partial least squares‐discriminant analysis
VIP: variable importance in projection

## 1 INTRODUCTION

At present, various ripening agents are being used in the process of fruit ripening. The variations in the ripening process of fruit not only affect the fruit quality but also associated with several health concerns such as hormonal imbalance, cerebral edema, reduced immunity, hypoxia, hematological and biochemical parameter changes, and increased susceptibility to cancers.^1^, ^2^, ^3^, ^4^ Different ripening practices/agents are used mainly for fruits like mango, papaya, banana, plums, etc.^5^ Ripening practices are used mainly to improve the appearance by maintaining uniformity in ripening and delivery of fruit for human consumption. However, these accelerated ripening processes may alter the fruit metabolism and fruit quality due to varying physiological processes influenced by multiple biological and environmental factors. Efforts to understand these aforesaid issues are very scanty.

Papaya (*Carica papaya* Linn.) is one of the highly metabolically active fruit that undergo significant changes during ripening. Papaya is also known as pawpaw that belongs to *Caricaceae* family.^6^ The production of papaya (broadly cultivated fruit) (11.22 Mt) is ranked the third after mango (38.6 Mt) and pineapple (19.4 Mt) around the world.^7^ India is placed at the first place in papaya production (∼ 5.3 Mt) followed by Brazil (1.5 Mt).^8^ Within India, Andhra Pradesh state is the largest producer of papaya with 30.7% of country's total production followed by Gujarat (22.1%)^9^ and is being abundantly consumed due to its health benefits.

Apart from nutritive benefits, papaya has a wide range of applications in therapeutics to improve the human health by reducing the risk of occurrence of various diseases.^10^ The alarming usage of various ripening practices/agents and their harmful effects on the fruit quality necessity an urgent need to understand the metabolic perturbations in papaya and their consequences on nutritional quality of the fruit.

Metabolomics is a comprehensive tool to understand the perturbations in the metabolism of food^11^ and biological systems that enable the detection of metabolic alternations in biological/physiological pathways influenced by genetic and environmental factors. Metabolomics using modern analytical platforms like gas chromatography‐mass spectrometry (GC‐MS), liquid chromatography‐mass spectrometry, and nuclear magnetic resonance for identification of low molecular weight metabolites. Among them, the more reliable, reproducible, user friendly that gives straightforward results on peak identification is GC‐MS based metabolomics approach.^12^ The GC‐MS–based metabolomics has advantages due to availability of mass spectral libraries and use of capillary columns makes it a valuable technique for studying the changes in metabolic profile of foods.

Over the past decade, the use of metabolomics emerged in the field of food science with increasing interest towards nutritional composition/quality, safety, and processing of food. Among all food groups, a fruit is an excellent source of metabolites as they possess complex physiological networks and various nutrients. The nutritive components are greatly influenced during the development and ripening process, which determines the quality of fruit. There are few studies that have focused on strawberry and peach metabolic profiling during development and ripening to determine their fruit quality.^13^, ^14^ In recent times, metabolomics play a significant role in understanding the postharvest physiological changes during ripening with different ripening practices.^15^, ^16^ Currently, about 15 ripening agents are available in the market to hasten the ripening process and among them ethylene and calcium carbide (CaC_2_) are most commonly used ripening agents.^17^ The use of CaC_2_ for ripening of fruit is stated as carcinogenic and prohibited by Food Safety and Standard Regulations 2011 (regulation 2.3.5), but still being used due to its low cost in comparison to ethylene.^18^ However, very less is known about the effects of these ripening agents on fruit quality. Thus, it is essential to investigate the metabolic changes occurring due to use of different ripening agents. The present work aims to use the GC‐MS–based metabolomics to understand the perturbations in papaya metabolome due to calcium carbide, ethylene, and room temperature ripening.

## 2 MATERIALS AND METHODS

### 2.1 Chemicals and reagents

Solvents of *n*‐hexane, methanol, and acetone of HPLC grade were purchased from Merck, and other derivatization reagents such as *o*‐ methoxyamine hydrochloride, pyridine, and *N*‐methyl‐*N*‐(trimethylsilyl) trifluoroacetamide at the purity of ≥98% were procured from Sigma Aldrich (St. Louis, MO, USA). The technical‐grade calcium carbide was procured from a local vendor at Hyderabad, India. All the chemicals and reagents used were of analytical grade.

### 2.2 Instrument

The GC‐MS system consisted of a 7890B gas chromatograph with a 5977A mass spectrometer in electron ionization mode (Agilent Technologies, Palo Alto, CA, USA). The chemstation software was used for the instrumental data acquisition, metaboanalyst (version 4.0) software was used for multivariate analysis (principal component analysis [PCA] and partial least squares‐discriminant analysis [PLS‐DA]), and MetPA software was used for pathway analysis.

### 2.3 Sampling and extraction

Nine papaya samples (green hard mature stage) were collected from orchards after examining the visual skin color as faint yellow stripe.^19^, ^20^ The collected samples were transported to the laboratory and divided into three groups. The samples were kept for 5 days under the ripening conditions as stated below. One sample group was kept at room temperature (30 ± 2°C) (termed as control), the second group was exposed to ethylene (100 μL^−1^) in a chamber (10 ft × 10 ft) at 16 ± 2°C for 24 h with relative humidity 90‐95% (subsequently stored at 24 ± 2°C), and the third group was in a corrugated carton box (16 inch × 12 inch) exposed to calcium carbide (1 g kg^−1^) wrapped in a newspaper and kept at the bottom of the fruits in a box at 30 ± 2°C and covered properly to prevent the leakage of acetylene gas. After 5 days, the samples were processed by separating the seeds and mashed the whole fruit to obtain a homogeneous fruit sample through grinding. The resultant sample was kept in liquid nitrogen in order to arrest the metabolic reactions and then was stored at −80 °C until further analysis.

For the analysis, 100 mg of papaya fruit sample was taken in an eppendorf tube and to this was added 500 μL of 80% methanol in ice cold condition.^16^ The resultant mixture was vortexed for 15 min at 4°C and then was centrifuged. The residue after decanting the supernatant was again extracted twice by repeating the same procedure. The resultant supernatants were pooled and centrifuged at 10 000 rpm for 10 min at 4°C and stored at −20°C until further analysis. An aliquot of 100 μL was taken in a separate vial and evaporated the solvent to dryness in a vacuum dryer for 5‐6 h (Scan Speed 32, Labogene, Denmark). The resultant extract was taken for derivatization before GC‐MS analysis.

### 2.4 Derivatization

Derivatization is an important step in GC‐MS metabolomics studies as many of the metabolites are polar in nature. The derivatization will convert them into nonpolar amenable for GC‐MS analysis and also helps to improve the volatility, suitability, efficiency, and detectability of the polar metabolites.

Prior to derivatization, the dried extract was mixed with 50 μL of methoxyamine hydrochloride (20 mg mL^−1^) in pyridine and incubated for 30 min at 60°C. This will help to stabilize the sugars in the samples by preventing the intramolecular conversions (hemiacetal to acetal) during derivatization. Further, an amount of 100 μL of MSTFA with 1% N‐Methyl‐N‐(trimethylsilyl)trifluoroacetamide (TMCS) was added to the resultant solution. The mixture was incubated at 60°C for 60 min in a thermomixer (Thermomixer C, Eppendorf AG 5382, Germany). After derivatization, the sample was made up to 600 μL with *n*‐hexane and vortex well prior to GC‐MS analysis.

### 2.5 GC‐MS analysis and screening of metabolites

The GC‐MS analysis was carried out with the following conditions reported by Ratnasekhar et al,^21^ after minor modifications. Briefly, an aliquot of 200 μL of a derivatized sample was taken into an insert with bottom spring in an air tight screw capped GC‐MS vials. The analysis was performed by injecting an aliquot of 1 μL of each extract via splitless mode into GC‐MS equipped with HP‐5MS capillary column (30 m × 250 μm *i.d*. × 0.25 μm film thickness) and electron ionization source (70 eV) at 230°C. The quadrupole analyzer, inlet, and GC‐MS interface temperatures were set at 150, 260, and 290 °C, respectively. The solvent delay was kept for 6 min. The mass spectrometer was used in full scan mode in the mass range of 29‐600 (m/z). Helium was used as a carrier gas with a constant flow of 1.2 mL min^−1^, and the GC oven program with an initial temperature set at 65°C for 2 min and then increased to 180°C at a rate of 5°C min^−1^, and further increased to 225°C at a rate of 3°C min^−1^, and finally increased to 265°C at a rate of 6°C min^−1^ held for 15 min with a total run time of 61 min. The process blank samples were also prepared as mentioned above without using the papaya sample in it to monitor the instrumental performance and to avoid artefacts during the analysis. The blank sample was injected after every six injections of each sample. After analysis, the peaks were identified by matching the acquired mass spectra of each peak with mass spectral library available in the instrument (NIST version 08).

### 2.6 Data processing and multivariate analysis

The fragmented spectrum was subjected to deconvolution by AMDIS (automated mass spectral deconvolution and identification system) software to separate them into individual spectra. AMDIS sequentially processes the GC‐MS raw data files, which are acquired from instrumental (chemstation) software. The raw data files converted into netCDF (network common data form) and automated processing was done by using XCMS software (based on ion with *m/z*, retention time, and peak matching).^22^ The samples and factors (metabolites) were taken as rows and columns labels, and the file was converted into comma‐separated values (.csv) format and then used for further processing. The normalization of the data was carried through pareto scaling to make features more comparable. The Metaboanalyst software (v.4.0) was used for PCA, which is an unsupervised approach to convert the multidimensional data into two‐dimensional space without losing significant features. PLS‐DA was also performed, which is a supervised approach to further refine the discriminant metabolites between the sample groups. The significant factors responsible for discrimination in the samples were selected based on variable importance in projection (VIP) score greater than 0.9. The classification performance of the model obtained in this study was validated through area under receiver operating characteristics (AUROC). Further, the quality was assessed by measuring *R* ^2^ (goodness of fit) and *Q* ^2^ (goodness of prediction) via cross‐validation and permutation test were performed (*n* = 100). Finally, for intuitive visualization of discriminatory metabolites, a heat map was constructed and relative concentrations of the potential metabolites between sample groups were compared.

### 2.7 Pathway analysis

The pathway analysis was carried using MetPA to understand the insights underlying metabolic insult in papaya fruit under studied conditions for perturbed metabolites identified after PLS‐DA analysis. MetPA is a web‐based tool, which integrates the results obtained from the advanced methods such as enrichment and topology analysis for visualization.^23^ For an over‐representative and topology analysis, hypergeometric, betweenness centrality was applied as algorithms, respectively. *Arabidopsis thaliana* (thale cress) was selected as a “model plant,” since *Caricaceae* is comes under the order Brassicales, same as *Arabidopsis thaliana*. The present analysis uses KEGG (*Kyoto Encyclopedia of Genes and Genomes*) metabolic pathway libraries as the knowledge base.

## 3 RESULTS AND DISCUSSION

**FIGURE 1:**
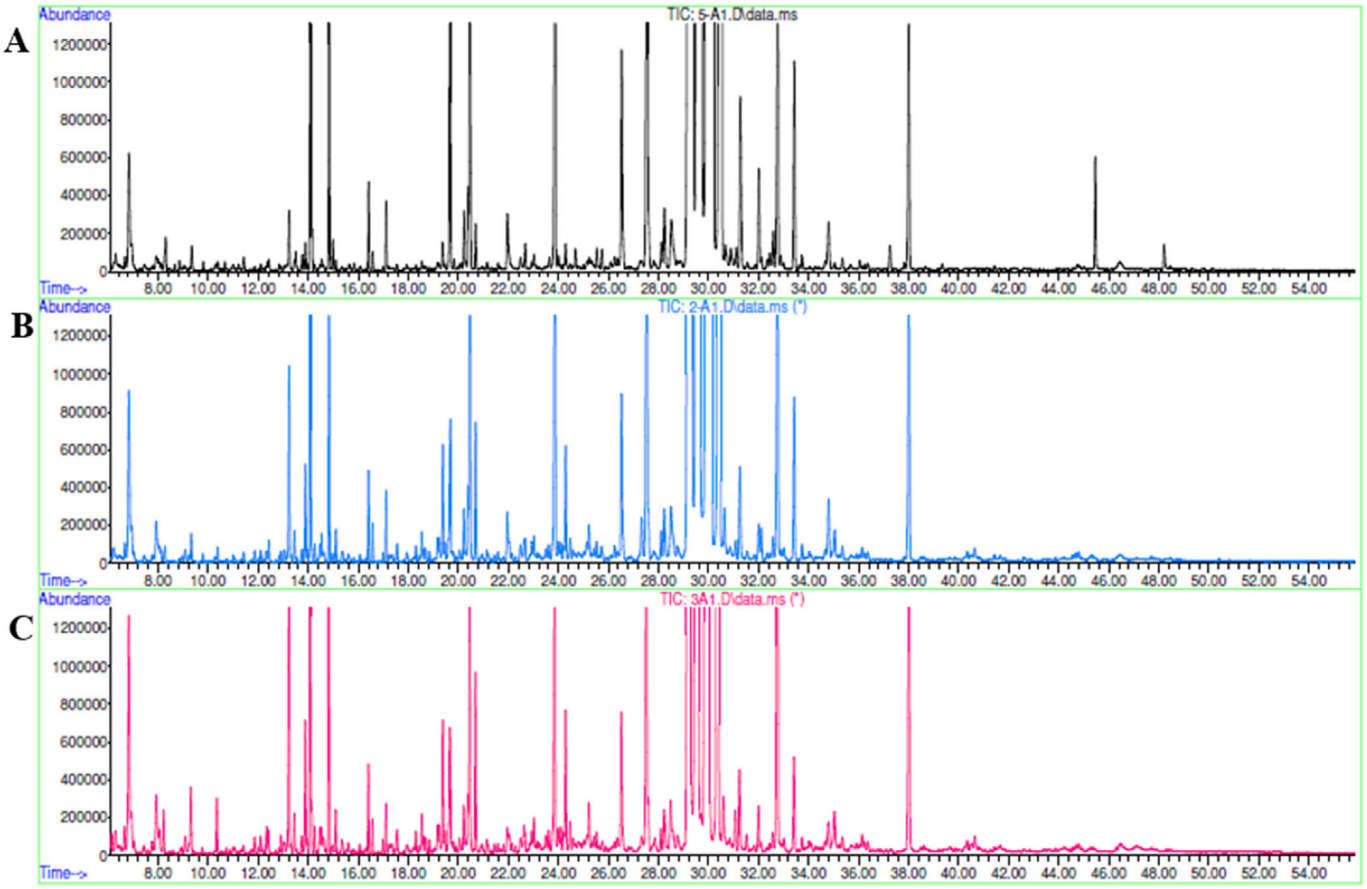
GC‐MS chromatogram for (A) control, (B) ethylene, and (C) CaC_2_ ripened fruit

After 5 days of ripening, all experimental groups of papaya fruit became softer and attains orange color (appropriate for consumption based on sensory observation) and this was stated as maturity stage 5.^19^ The metabolic profiling of papaya fruit with three different ripening practices/agents was performed using GC‐MS, and the majority of the peaks were common among all the groups. A total of 100 metabolites were identified from the acquired data by matching the spectra's of these metabolites with the mass spectral library (NIST 08) available in the instrument after deconvolution. The metabolites identified belong to the classes of amino acids (15), organic acids (19), fatty acids (27), sugars (14), polyols (5), and others (20) (Figure 1). It is difficult to draw the inferences from the multidimensional data (due to many variables of multiclass metabolites) and a large number of samples. So, chemometric analysis (unsupervised PCA and supervised PLS‐DA) was performed to reduce the dimensionality (multi to two) of the data without affecting the actual variations in the sample groups (control, ethylene, and calcium carbide samples) and to identify differential metabolites responsible for this classification.

**FIGURE 2:**
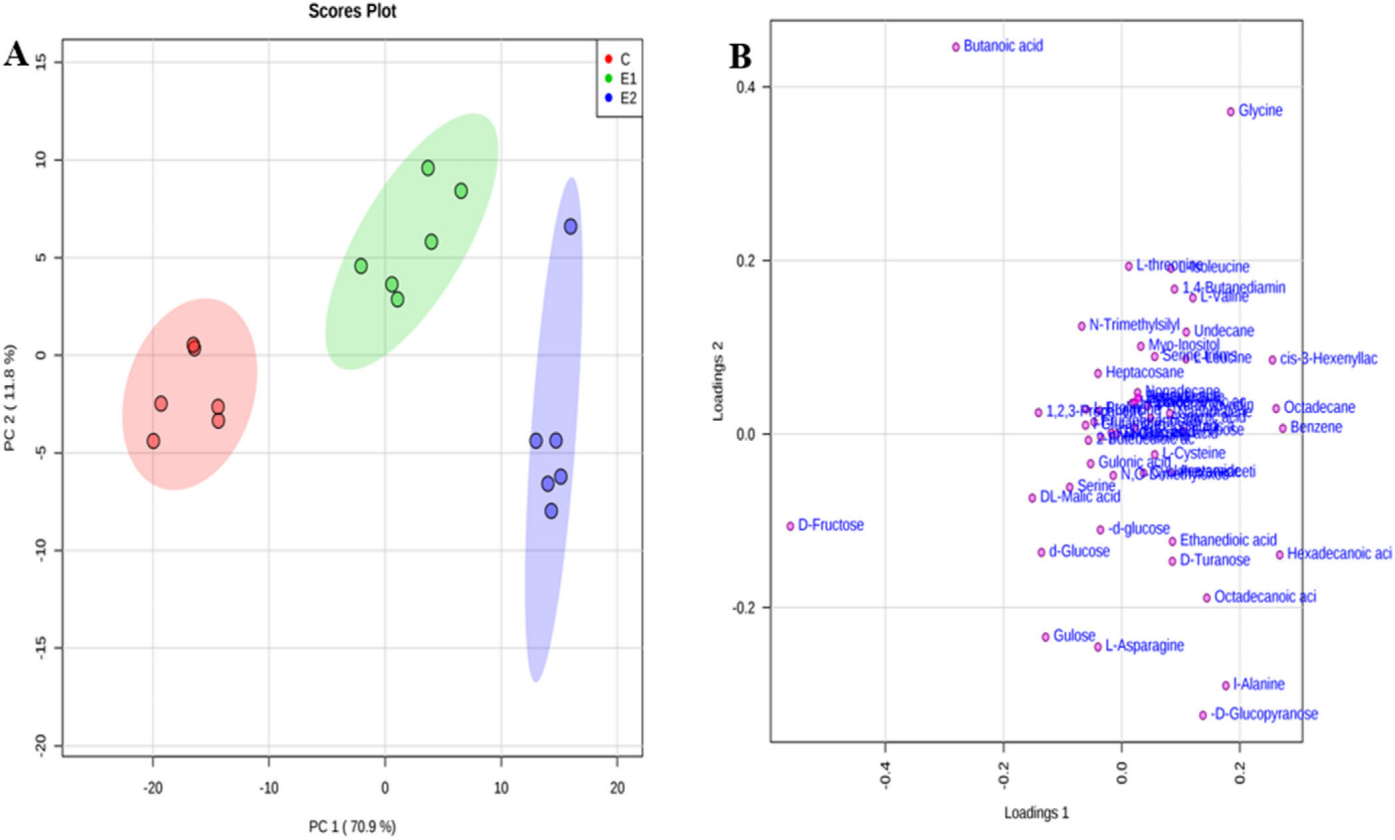
(A) PCA score plot indicating discrimination between control (C), ethylene (E1), CaC_2_ (E2) exposed samples after GC‐MS analysis. (B) Loading plot for all the three groups showing the metabolites that were major contributors to the separation of groups observed in the PCA score plot of papaya fruits

The unsupervised PCA will provide a classification pattern between the samples ripened with different practices/agents by plotting a pairwise score plots. The total accumulated variance accounted for 94.3% and variance explained individually like PC_1_ of 70.9%, PC_2_ of 11.8%, PC_3_ of 6%, PC_4_ of 3.4%, and PC_5_ of 2.4%. The first two components PC_1_ and PC_2_ accounted for the maximum variability of about 82.7%. The clear separation of three different clusters, namely control (C), ethylene ripened (E1), and CaC_2_ ripened (E2) papaya in PCA trajectory score plot, clearly indicates the metabolic perturbations in the metabolome of papaya which is specific to the ripening practices/agents (Figure 2A). The corresponding loading plot (Figure 2B) outlines the metabolites responsible for the classification of these sample groups in the score plot.

In total, 20 metabolites including sugars (d‐fructose, d‐glucopyranose, d‐glucose, gulose, d‐turanose), amino acids (l‐alanine, glycine, l‐valine, threonine, leucine), organic acids (dl‐malic acid, 1,2,3‐propane tricarboxylic acid, butanoic acid, benzene, *cis*‐3‐hexenyllactate), and fatty acids (hexadecanoic acid, octadecanoic, octadecane, ethanedioic acid) were found in the samples.

**FIGURE 3:**
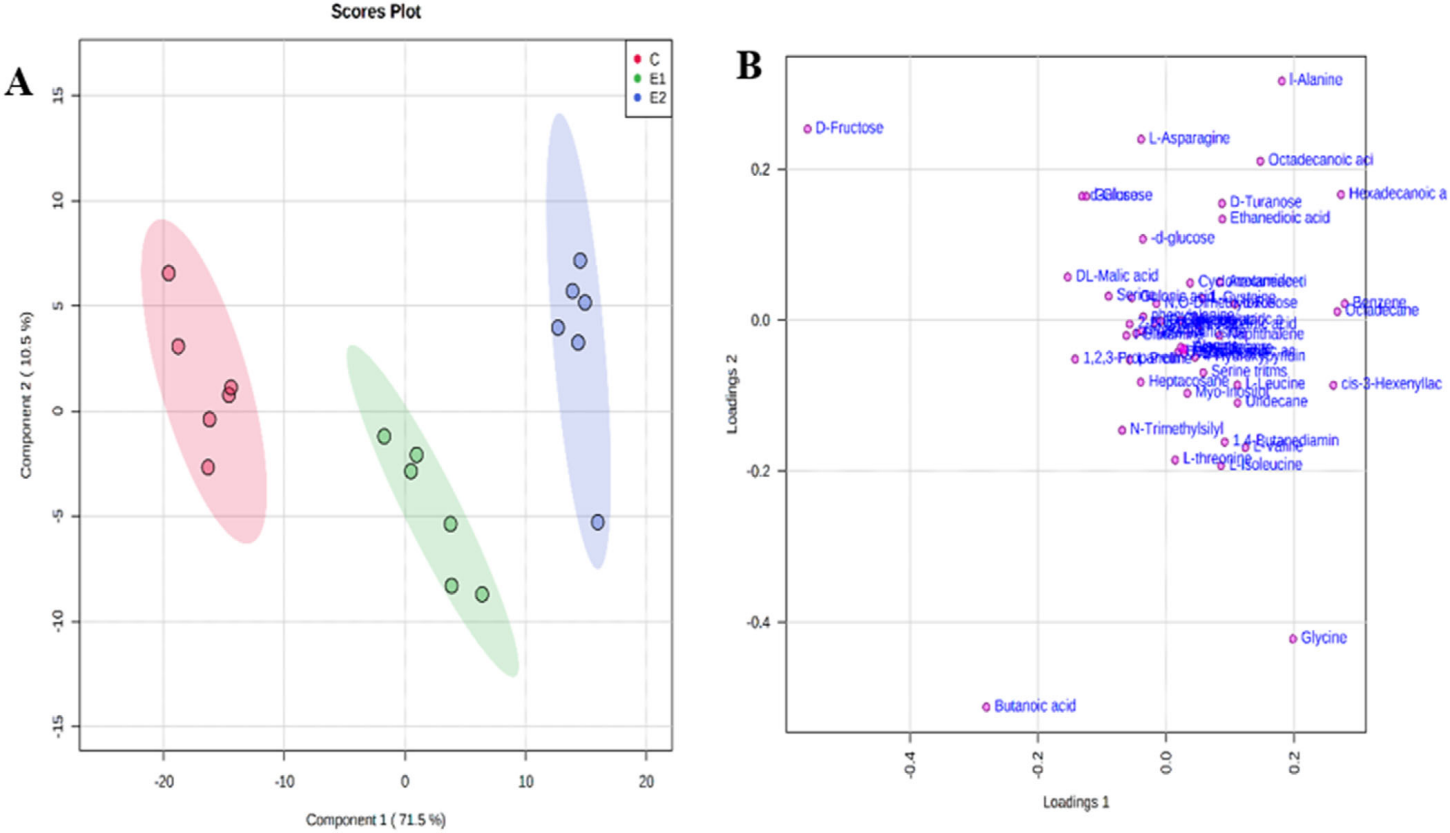
(A) PLS DA score plot and (B) loading plot

**FIGURE 4:**
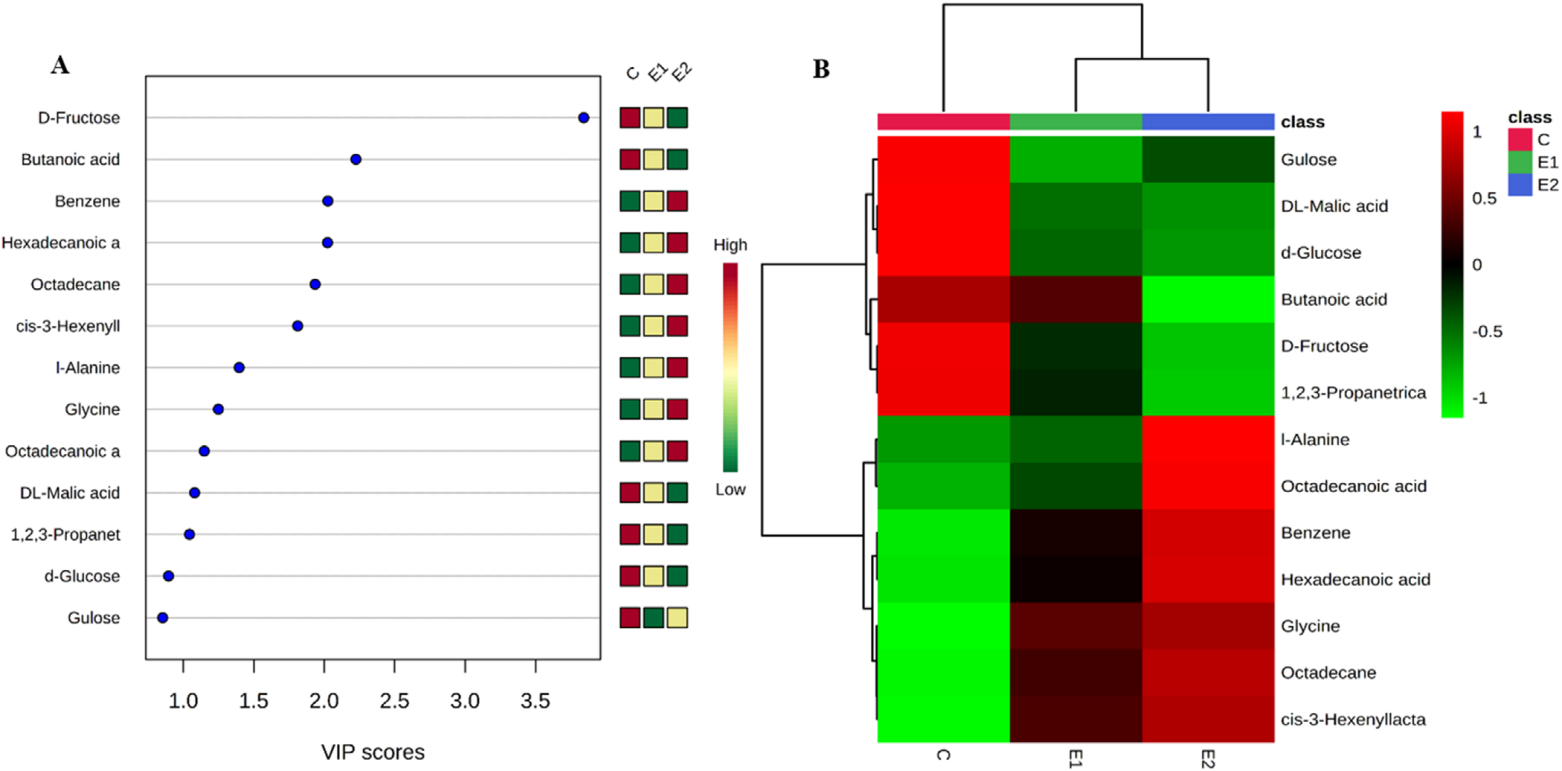
(A) VIP score plot and (B) heat map for 13 metabolites selected from the VIP score plot by PLS‐DA analysis

Then to refine further the results of classification obtained after the unsupervised PCA approach, the supervised PLS‐DA was used to generate a score plot and its corresponding loading plot from intergroup comparison of each group verses control (Figure 3A and 3B). Further screen to identify the differential metabolites through a statistical parameter called the VIP score. The results showed that 13 metabolites were significantly altered in papaya due to the use of different ripening practices/agents and identified them as candidate marker metabolites for this study (Table 1; Figure 4A). The metabolites, namely d‐fructose, butanoic acid, benzene, hexadecanoic acid, octadecane, *cis*‐3‐hexenyllactate, l‐alanine, glycine, octadecanoic acid, dl‐malic acid, 1,2,3‐propanetricarboxylic acid, d‐glucose, and gulose, were identified as candidate marker/differential metabolites.

**TABLE 1:** Perturbed metabolites in papaya fruit metabolome after exposed to different ripening practices/agents as identified by GC‐MS–based metabolomics

Metabolite	Molecular formula	VIP score	Fragmentation pattern (*m/z*)	*RT* (min)	*P value	Significance variance (CaC_2_ vs Control)	Significance Variance (EthyleneVs Control)
d‐Fructose	C_21_H52O_6_Si_5_	3.89	43, 57,73, 85147 217, 437	27.31	7.61E−04	Down	Down
*cis*‐3‐Hexenyl lactate	C_9_H_16_O_3_	1.77	43, 55, 67, 82, 89	6.82	6.27E−04	Up	Up
l‐Alanine	C_9_H_23_NO_2_Si_2_	1.37	45, 73, 100, 116, 190	9.3	.00784	Up	Up
Benzene	C_14_H_22_	1.99	41, 57, 91, 115, 147, 175, 190	13.23	5.11E−04	Up	Up
Glycine	C_7_H_12_F_3_NO_3_Si	1.15	42, 58, 73, 110, 134, 184, 228, 285	12.34	.00812	Up	Up
dl‐Malic acid	C_13_H_30_O_5_Si_3_	1.07	55, 73, 133, 147, 189, 233	19.64	0.00189	Down	Down
1,2,3‐Propane tricarboxylic acid	C_18_H_40_O_7_Si_4_	1.04	45, 73, 147, 211, 273, 347, 363, 465	27.51	7.74E−04	Down	Down
Hexadecanoic acid	C_25_H_54_O_4_Si_2_	1.99	43, 57, 73, 103, 129, 147, 239	45.45	5.11E−04	Up	Up
Octadecanoic acid	C_27_H_58_O_4_Si_2_	1.13	43, 57, 73, 129, 147, 399, 487	48.21	5.11E−04	Up	Up
Octadecane	C_19_H_40_	1.91	43, 57, 71, 85, 99, 113, 127, 141, 155	24.28	5.11E−04	Up	Up
Butanoic acid	C_13_H_33_NO_2_Si_3_	2.24	45, 73, 86, 100, 147, 174, 246, 304	20.46	.00168	Down	Down
d‐Glucose	C_22_H_55_NO_6_Si_5_	0.946	73, 89, 103, 117, 157, 189, 205, 244, 319	28.21	.01084	Down	Down
Gulose	C_21_H_52_O_6_Si_5_	0.9	73, 103, 129,147, 204, 220, 319	33.42	.01796	Down	Down

*Significance levels was calculated from nonparametric version ANOVA.

The individual comparisons of calcium carbide with control samples has resulted in alternations in d‐fructose, butanoic acid, benzene, hexadecanoic acid, octadecane, *cis*‐3‐hexenyllactate, l‐alanine, glycine, octadecanoic acid, dl‐malic acid, 1,2,3‐propanetricarboxylic acid, d‐glucose, gulose, and all 13 metabolites were found to be common with PLS‐DA of multiple groups (control vs calcium carbide vs ethylene). In the same way, the individual comparison of ethylene ripening with controls samples has resulted in alternations in d‐fructose, octadecane, gulose, *cis*‐3‐hexenyllactate, benzene, hexadecanoic acid, glycine, dl‐malic acid, 1,4‐butanediamine, l‐isoleucine, d‐glucose, l‐valine, 1,2,3‐propanetricarboxylic acid, butanoic acid, undecane, out of which, 11 metabolites (d‐fructose, octadecane, gulose, *cis*‐3‐hexenyllactate, benzene, hexadecanoic acid, glycine, dl‐malic acid, d‐glucose, 1,2,3‐propanetricarboxylic acid, butanoic acid) were found to be common with PLS‐DA of multiple groups (control vs calcium carbide vs ethylene). This shows the predictive accuracy of the data by PLS‐DA. The heat map showed the relative abundance for differential metabolites in the experimental groups (control vs ethylene vs calcium carbide) was represented in colors (red‐high abundance, green‐low abundance) with a scale expressed value (−1 to 1) (Figure 4B).

**FIGURE 5:**
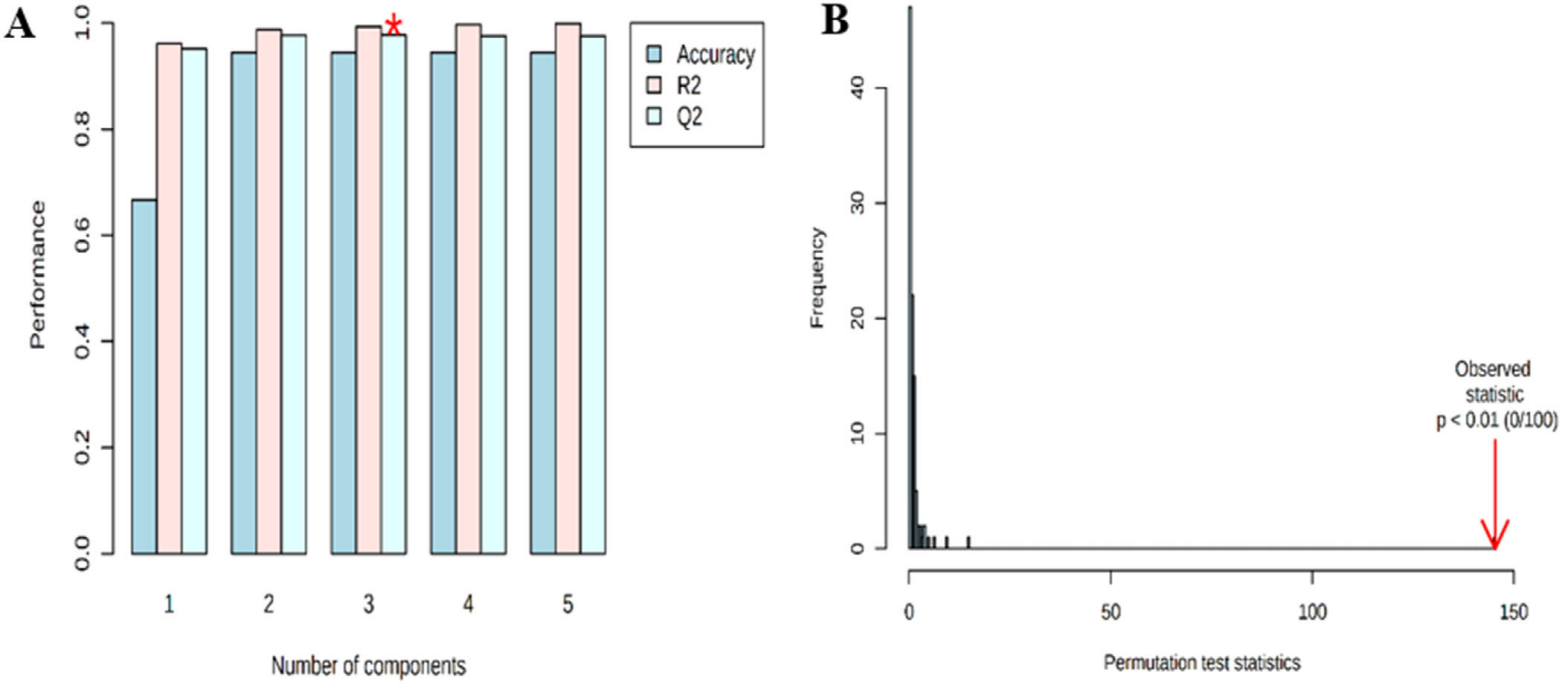
(A) Cross‐validation, bar plots showing the quality measures (accuracy, *Q* ^2^, *R* ^2^). (B) Permutation analysis of PLS‐DA models derived from artificially ripened and normal ripened papaya fruit

To validate the predictive power of the classification given by these approaches and to know about the quality of the model obtained, we have further performed the cross‐validation by the leave‐one‐out cross‐validation method and the current data show the predictive accuracy *Q* ^2^ ˃ 0.97 and *R* ^2^ ˃ 0.96 and it represents a good fit of the model (Figure 5A).

**FIGURE 6:**
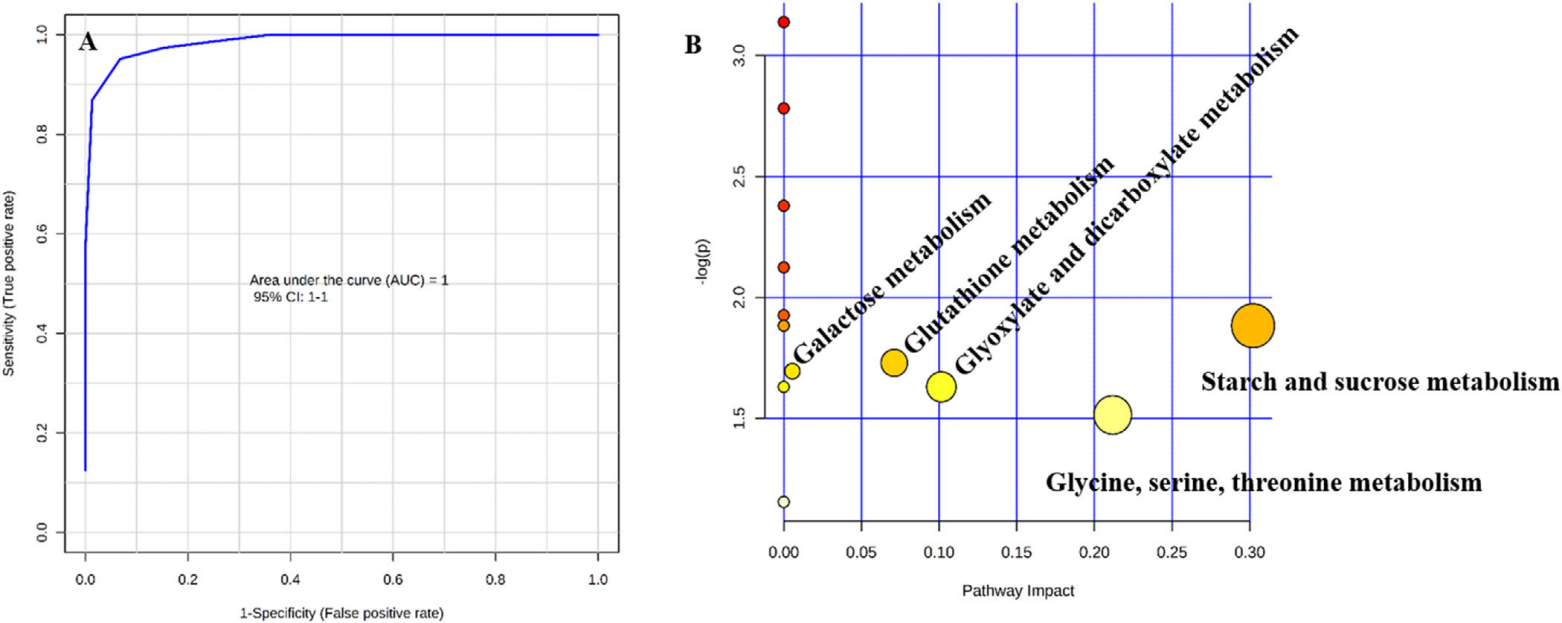
(A) ROC curve analysis (AUROC = 1) based on cross‐validation performance. (B) Summary of path way analysis. *x*‐Axis represents the pathway impact value (node radius represents the impact value) by topology analysis, and *y*‐axis represents different levels of significance (node color indicates the *p* value, yellow to red) by enrichment analysis

Further, the permutation test has also been performed for model validation and determined that the *P*‐value as ˂ .001 with a permutation number setting it as 100 (Figure 5B). Apart from the above, classification performance of these models was evaluated by a ROC curve plotted between sensitivity (true positive rate) vs 1‐ specificity (false positive rate) and AUROC was found to be 1.0, which is considered to be excellent classification performance of the model from this study (Figure 6A).

dl‐Malic acid said to be one of the prominent organic acid found to have upregulated in control than that of ethylene and CaC_2_‐ripened papaya fruit. The other acid known as 1,2,3‐propane tricarboxylic acid (tricarballylate), which is said to be a major inhibitor of aconitase in Kreb's cycle,^24^ was detected in papaya and found to be significantly higher in the control group than in ethylene‐ and CaC_2_‐ripened papaya fruit. Thus, we can assume that an increased concentration of 1,2,3‐propane tricarboxylic acid could be an index for accumulation of citric acid, indicating the possible decreased acidity in ethylene‐ and CaC_2_‐ripening papaya fruit.^25^ Similarly, the sugars like d‐fructose, d‐glucose, and gulose were found to be increased in control than in ethylene‐ and CaC_2_‐ripened papaya. The decreased levels of sugars in ethylene‐ripened papaya fruit may be due to the storage at lower temperature, which causes the reduced rate of reaction which in turn impairs starch metabolism.^26^ The reduced levels of sugars in CaC_2_‐ripened papaya may depend on the distinct rate of synthesis and degradation of sucrose or levels of polysaccharide content and their subsequent conversion to reducing sugars^27^ in the course of rapid ripening or utilization of sugars as an energy source for ripening of the fruit and are in line with the literature findings on mango ripening with CaC_2_.^28^, ^29^ Benzene, a volatile aromatic hydrocarbon found in the horticulture crops is the strongest evidence of carcinogenicity. In the present study, the levels of benzene were higher in CaC_2_‐ripened fruit as compared to ethylene and control and was higher in ethylene‐ripened fruit as compared to control papaya fruit. These findings clearly suggest that the benzene accumulation in CaC_2_‐ripened papaya might be an indication for the presence of hydroxyl radicals induced decarboxylation or decomposition of amino acids.^30^, ^31^, ^32^

*cis*‐3‐Hexenyl lactate is said to be an ester of lactic acid and normally found in the fruit during the esterification reaction in the presence of lipase,^33^ this ester has a characteristic flavor and found to be higher in CaC_2_‐ripened fruit in comparison to control and ethylene‐ripened papaya fruit. The levels of amino acids, namely l‐alanine and glycine, were upregulated in the CaC_2_‐ripened papaya fruit, which may be attributed to the presence of impurities in CaC_2_,^34^ and contribute effectively in the defense mechanism to detoxify the xenobiotics from the fruit.^35^ Further, free amino acids are precursors of aroma compounds during ripening.^36^

Octadecane is said to be a volatile compound and found to be one of the main flavored compounds in papaya.^37^ Generally, several biochemical or metabolic pathways were responsible for release of a mixer of volatile compounds from fruits which reflect the quality attribute like fruit flavor. In the present study, a considerable increase of octadecane in the CaC_2_‐ripened papaya fruit was observed as compared to other two groups. This increase might reflect the advanced ripening in the CaC_2_‐ripened fruit compared to that of ethylene and control. But until now, we have a limited understanding how volatile compounds influence the flavor quality upon ripening.^38^

In the present study, an increase in butanoic acid levels was observed in control fruit and it may be associated with off‐flavor. Previous studies have also reported that the increased butanoic acid was responsible for undesirable flavor as a consequence of storage.^36^ In contrast, the decreased levels of butanoic acid were observed in the fruit when exposed to ethylene and CaC_2_ in comparison to control samples. Further, compounds like hexadecanoic acid and octadecanoic acid were found to be upregulated in the CaC_2_‐ripened papaya fruit as compared to ethylene and control ripened fruit. The fatty acids are said to be increased during fruit ripening and correlates with aroma and flavor of the fruit.^39^ The increase in these fatty acids was high in the CaC_2_‐ripened papaya and a moderate increase in the ethylene‐ripened papaya fruit than in comparison to control papaya. This may be attributed to the fastening of the ripening, which is in the order of CaC_2 _> ethylene > control ripening process.

### 3.1 Pathway analysis

**FIGURE 7:**
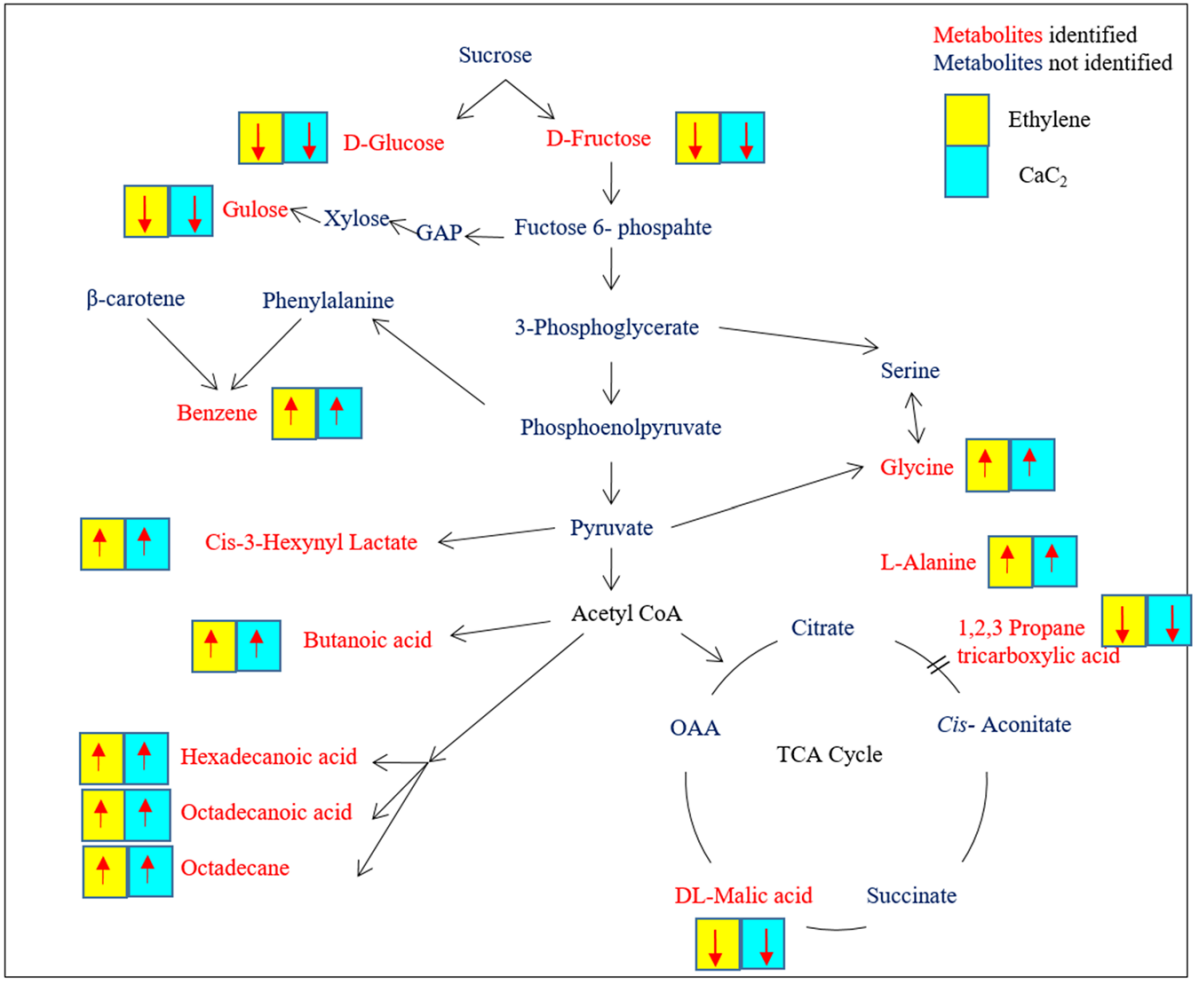
Schematic representation of metabolic pathways affected due to different ripening process/agents in papaya fruit. Red color metabolites were identified blue color metabolite were nonidentified. ↑ significant increase/upregulated, ↓ significant decrease/downregulated with chemical exposure (ethylene, CaC_2_) with respect to control was mentioned. Significance levels were calculated from nonparametric version ANOVA

The present study has shown that the changes in metabolome of papaya were significant with different ripening practices/agents. The study also examined the affected metabolic pathways in papaya samples due to use of different ripening agents/practices. The pathways include starch and sucrose metabolism, glycine, serine, and threonine metabolism, Glyoxylate and dicarboxylate metabolism, glutathione metabolism, and galactose metabolism were affected as identified by MetPA (Figure 6B). The differential metabolites in the pathways altered are shown in Table 2. Further, from the information obtained after the chemometric analysis, the possible pathway interaction map was illustrated to identify the differential metabolites identified in each pathway (Figure 7).

**TABLE 2:** Altered metabolites showing an impact on respective metabolic pathways

Identified pathways by using KEGG library	Matched metabolites	−log(*p*)	Impact	FDR
Starch and sucrose metabolism	d‐Glucose	1.88E+00	0.3	1.00E+00
Glycine, serine, and threonine metabolism	Glycine	1.69E+00	0.21	1.00E+00
Glyoxylate and dicarboxylate metabolism	Glycine	1.81E+00	0.1	1.00E+00
Glutathione metabolism	Glycine	1.91E+00	0.07	1.00E+00
Galactose	d‐Glucose	1.69E+00	0.005	1.00E+00

## 4 CONCLUDING REMARKS

In the present study, we made an attempt to understand the metabolic perturbations occurring due to different ripening practices/agents used to ripe *Carica papaya* L. by the GC‐MS–based metabolomics approach. The present study identified 13 metabolites, which were significantly altered due to different ripening practices/agents. The amino acids, organic acids, and fatty acids along with sugars were significantly altered their levels, and some of them were specific to the ripening practices/agents. The study also identified that five metabolic pathways were significantly affected during the ripening practices/agents. Though the ripening agents (ethylene and CaC_2_) impart the ripened look for fruits, but there was a difference in certain metabolites and respective pathways related to taste and aroma when compared to control. The GC‐MS–based metabolomics approach can provide vital information to identify candidate marker metabolites in understanding the fruit‐ripening processes, which eventually help in assessing the food quality, safety, and biotechnological aspects. However, more research needs to be conducted to explore the detailed mechanism with different batches of fruit in different maturity stages for a longer time.

## FUNDING SOURCE

UGC New Delhi to Mr. Hussain Shaik for Senior Research Fellowship

DST, New Delhi through Grand‐in‐Aid project (GAP‐0701)

## CONFLICT OF INTEREST

The authors have declared no conflict of interest.
